# Some Points in the Anatomy, Pathology, and Surgery of Intussusception

**Published:** 1898-12

**Authors:** 


**Some Points in the Anatomy, Pathology, and Surgery of Intussusception.** By D'Arcy Power, M.B. Pp. 88. London: The Rebman Publishing Company, Limited. 1898.

Mr. D'Arcy Power issues in an enlarged form the lectures which he delivered at the College of Surgeons in February, 1897. The book is divided into three chapters, the first one being devoted to the morbid histology of the different stages of intussusception, the second to the pathology, and the third to treatment.

Much labour and original work have been expended over the first chapter, sections having been cut of numerous specimens, both recent and old, from the various hospital museums; and Mr. Power shows us that the greatest changes take place in the submucous tissue, which becomes congested, hemorrhages taking place into its structure, and that it becomes eventually inflamed.

An interesting point is the increase in the number of goblet cells, which may account for the excessive amount of mucus produced in these cases.

The second chapter opens with an account of the anatomy of the ileo-cæcal region, and there is an elaborate table, comprising 64 examinations of infants and small children, dealing mainly with the relationship of the ileum to the cæcum as to size, direction of axis, and arrangement of peritoneal folds. It is shown that, compared with adults, the cæcum is in infants relatively larger than the ileum, which may be one determining cause of intussusception at this point.

When we come to the last chapter, we cannot help feeling that Mr. Power's labours in elucidating the pathology of this affection have not done much towards advancing the treatment. He thinks irrigation *may* be tried in those acute cases which have not lasted longer than forty-eight hours; that in cases of longer standing than this, in those in which after reduction by irrigation the intussusception has returned three times, and in those in which there is abundant hemorrhage, laparotomy must be performed. If the bowel cannot then be all reduced, it must be dealt with according to the individual surgeon's experience and the appliances at hand.

